# A Wide Spectrum of Autoimmune Manifestations and Other Symptoms Suggesting Immune Dysregulation in Patients With Cartilage-Hair Hypoplasia

**DOI:** 10.3389/fimmu.2018.02468

**Published:** 2018-10-25

**Authors:** Svetlana Vakkilainen, Riikka Mäkitie, Paula Klemetti, Helena Valta, Mervi Taskinen, Eystein Sverre Husebye, Outi Mäkitie

**Affiliations:** ^1^Children's Hospital, University of Helsinki and Helsinki University Hospital, Helsinki, Finland; ^2^Folkhälsan Research Center, Helsinki, Finland; ^3^University of Helsinki, Helsinki, Finland; ^4^Department of Clinical Science, University of Bergen, Bergen, Norway; ^5^K.G. Jebsen Centre for Autoimmune Disorders, University of Bergen, Bergen, Norway; ^6^Department of Medicine, Haukeland University Hospital, Bergen, Norway; ^7^Department of Molecular Medicine and Surgery, Karolinska Institutet and Clinical Genetics, Karolinska University Hospital, Stockholm, Sweden

**Keywords:** allergy, asthma, autoantibodies, enteropathy, *RMRP*

## Abstract

**Background:** Mutations in *RMRP*, encoding a non-coding RNA molecule, underlie cartilage-hair hypoplasia (CHH), a syndromic immunodeficiency with multiple pathogenetic mechanisms and variable phenotype. Allergy and asthma have been reported in the CHH population and some patients suffer from autoimmune (AI) diseases.

**Objective:** We explored AI and allergic manifestations in a large cohort of Finnish patients with CHH and correlated clinical features with laboratory parameters and autoantibodies.

**Methods:** We collected clinical and laboratory data from patient interviews and hospital records. Serum samples were tested for a range of autoantibodies including celiac, anti-cytokine, and anti-21-hydroxylase antibodies. Nasal cytology samples were analyzed with microscopy.

**Results:** The study cohort included 104 patients with genetically confirmed CHH; their median age was 39.2 years (range 0.6–73.6). Clinical autoimmunity was common (11/104, 10.6%) and included conditions previously undescribed in subjects with CHH (narcolepsy, psoriasis, idiopathic thrombocytopenic purpura, and multifocal motor axonal neuropathy). Patients with autoimmunity more often had recurrent pneumonia, sepsis, high immunoglobulin (Ig) E and/or undetectable IgA levels. The mortality rates were higher in subjects with AI diseases ($\chi_{\left(2\right)}^{2}$ = 14.056, *p* = 0.0002). Several patients demonstrated serum autoantibody positivity without compatible symptoms. We confirmed the high prevalence of asthma (23%) and allergic rhinoconjunctivitis (39%). Gastrointestinal complaints, mostly persistent diarrhea, were also frequently reported (32/104, 31%). Despite the history of allergic rhinitis, no eosinophils were observed in nasal cytology in five tested patients.

**Conclusions:** AI diseases are common in Finnish patients with CHH and are associated with higher mortality, recurrent pneumonia, sepsis, high IgE and/or undetectable IgA levels. Serum positivity for some autoantibodies was not associated with clinical autoimmunity. The high prevalence of persistent diarrhea, asthma, and symptoms of inflammation of nasal mucosa may indicate common pathways of immune dysregulation.

## Introduction

Cartilage-hair hypoplasia (CHH, OMIM #250250) is caused by mutations in *RMRP*, which encodes the long non-coding RNA component of mitochondrial RNA-processing endoribonuclease. CHH is characterized by metaphyseal chondrodysplasia, hair hypoplasia, combined immunodeficiency, anemia, and increased risk of malignancies. Autoimmune (AI) diseases have occasionally been reported in CHH, such as enteropathy, hemolytic anemia (AIHA), hypoparathyroidism, hypo- or hyperthyroidism, juvenile rheumatoid arthritis and neutropenia (1–4). The prevalence of asthma is increased and has been reported in 16% (4/25) of Amish and 24% (10/34) of Finnish patients (3, 5). Allergic rhinoconjunctivitis is also excessively prevalent (41%, 23/56) in the Finnish CHH population (6).

*RMRP* mutations induce cell cycle abnormalities resulting in defective proliferation and increased apoptosis of T cells (7, 8). Patients with CHH have markedly reduced numbers of recent thymic emigrants and low naïve CD4+ and CD8+ cells, but normal counts of CD4+CD25_high_CD127_low_ regulatory T cells and double-negative T cells (6, 8). Eosinophilia and increased immunoglobulin (Ig) E levels are uncommon (6).

The knowledge of the disease mechanisms in CHH has expanded in recent years to include impaired gene regulation (9) and defects in telomere biology (10, 11). In addition, lack of autoimmune regulator (AIRE) expression and absence of Foxp3+ T cells in the thymus of a single patient with CHH has been reported (12). The loss of AIRE function associated with *AIRE* mutations is responsible for Autoimmune Polyendocrinopathy-Candidiasis-Ectodermal Dystrophy (APECED), a recognized polyendocrine syndrome with defective T and B cell tolerance (13, 14). This could support a possible role of impaired regulatory T cell function in the pathogenesis of CHH.

We have recently demonstrated broad autoantibody reactivity in subjects with CHH using the microarray technique (15). Compared with healthy controls, CHH patients showed significantly higher reactivity for autoantibodies against gliadin, nuclear antigens, thyroglobulin, vitronectin, as well as for myositis-specific antibodies.

In order to further explore the prevalence and characteristics of AI and allergic manifestations in individuals with CHH, we analyzed clinical and laboratory data for a large group of Finnish CHH patients. We also tested serum samples from several of these patients for a variety of autoantibodies. To complement our previous research, we applied different measurement methods and tested mostly for autoantibodies that were not included in our recent study (15).

## Methods

We originally invited all known Finnish patients with CHH to a prospective cohort study in 1985–1991. Those who agreed to participate (Cohort 1, *n* = 80) underwent interview, clinical examination and blood sampling; *RMRP* mutation testing was performed when the gene was identified in 2001. A follow-up visit was conducted in 2012–2015, when Cohort 1 and all other thus far identified Finnish CHH patients were contacted and invited for a clinical visit and blood tests. This study thus included three groups of patients (in total 104 patients): (1) patients from Cohort 1 who attended the follow-up visit (*n* = 32), (2) patients from Cohort 1 who were unavailable for the follow-up visit (*n* = 48), and (3) patients recruited to the study at the follow-up visit (Cohort 2, *n* = 24). We collected complete data by means of interviews and from hospital records for all patients participating the follow-up visit (*n* = 56). For those patients from Cohort 1 who did not attend the follow-up visit, we obtained health information from two Finnish National Medical Databases. The Care Register for Health Care has since 1969 recorded the activities of health centers, hospitals and other institutions providing inpatient care, as well as home-nursing care. Outpatient primary health care data was derived from the Register of Primary Health Care Visits, which covers all health centers in Finland since 2011. Database information included health service providers, dates of the visits, diagnoses, as well as diagnostic and therapeutic procedures. We then contacted all identified hospitals and requested all patients' detailed health records for the analysis. Special attention was given to the signs and symptoms attributed to autoimmunity, such as skin rashes, joint symptoms and gastrointestinal complaints and they were systematically discussed during the interviews.

Asthma diagnosis was made by physicians and/or pulmonologists using spirometry and assessment of response to asthma medications. Allergy was similarly diagnosed by physicians based on clinical presentation and in some cases, on skin tests and/or serum specific IgE levels. Sepsis diagnosis was made by physicians and supported by positive findings in blood cultures.

Serum samples for autoantibody measurement were obtained from those who agreed to donate blood at the follow-up visit (*n* = 33) and stored at −70°C until analyses. The analyzed autoantibodies were related to celiac disease, based on frequent gastrointestinal complaints among the patients, and on autoantibodies that are commonly seen in patients with *AIRE* mutations (Table 1). One of the eight chosen autoantibodies (deamidated gliadin peptide IgG) has previously been tested with microarray technique in 16 of the available 33 samples (15).

**Table 1:** Antibody assays performed on serum samples of patients with cartilage-hair hypoplasia.

**Antibody**	**Abbreviation**	**Measurement method**	Normal values in adults^*^
Deamidated gliadin peptide IgA	DGP-IgA	FEIA	<7 U/ml
Deamidated gliadin peptide IgG	DGP-IgG	FEIA	<7 U/ml
Tissue transglutaminase IgA	tTG-IgA	FEIA	<7 U/ml
Tissue transglutaminase IgG	tTG-IgG	FEIA	<10 U/ml
Endomysium IgA	EMA-IgA	IIF	Titer <5
Endomysium IgG	EMA-IgG	IIF	Titer <5
Interleukin 17 IgG	IL-17	Radioligand binding^1^	<218 Index
Interleukin 22 IgG	IL-22	Radioligand binding^1^	<270 Index
Interferon ω IgG	IFN-ω	Radioligand binding^2^	<200 Index
21-Hydroxylase IgG	21-OH	Radioligand binding^3^	<57 Index

*FEIA fluorometric enzyme immunoassay, Ig immunoglobulin, IIF indirect immunofluorescence*,

* *Age-specific institutional reference values were used to evaluate results in pediatric patients*.

1 *(16)*.

2 *(17)*.

3 *(18)*.

To further evaluate the cause for symptoms related to rhinoconjuctivitis we examined nasal samples for eosinophilic, neutrophilic and goblet cells from five patients with physician-diagnosed allergic rhinitis. Subjects had refrained from application of local nasal decongestants, steroids or systemic antihistamines prior to sampling. Samples were taken during spring and summer months (May–August), from both anterior nares beneath the middle turbinates using a standard cotton swab. Samples were let to set on a glass slide at room temperature. Cytology was evaluated by an experienced technician using eosin-methylene blue staining and microscopy.

Statistical analyses were performed with IBM SPSS version 23 software. Fisher's exact test, logistic regression analysis and Kaplan-Meier method were implicated when appropriate.

The study protocol was approved by the Institutional Research Ethics Committee at Helsinki University Hospital, Finland, and informed consents were obtained from all participants and caregivers.

## Results

Our study group included 104 patients (61 women, 43 men) with genetically confirmed CHH. Their median age at the last follow-up was 39.2 years (range 0.6–73.6 years). There were 17 children aged <18.0 years, 49 young adults (18.0–45.0 years) and 38 adults aged >45.0 years. The majority of patients (*n* = 79; 76%) were homozygous for the g.70A>G *RMRP* mutation, others were compound heterozygous for g.70A>G and either g.262G>T (*n* = 22; 21%) or duplication TACTCTGTGA at −13 (*n* = 3; 3%).

Altogether 11 patients (11/104, 10.6%) had been diagnosed with AI conditions (Table 2*)*. Clinical features of all study patients are presented in Table 3. In addition to AI diseases that have been previously reported in patients with CHH (enteropathy, hemolytic anemia, hyperthyroidism, and juvenile rheumatoid arthritis), we report psoriasis, idiopathic thrombocytopenic purpura, narcolepsy, and multifocal motor axonal neuropathy all of which have been regarded as autoimmune diseases (19–22).

**Table 2:** Autoimmune conditions diagnosed in 11 out of 104 patients with cartilage-hair hypoplasia.

**No**.	**AI disease**	**Age at diagnosis of AI disease, yrs**	**Age at the latest follow-up, yrs**	**Treatment**	**Outcome of AI disease**	**Other symptoms**
1	Enteropathy	3	Died in young adulthood	None specific	Died from pneumonia following bowel occlusion	Anemia, arthralgia, asthma, pneumonia
	MMAN	28		IVIG	Moderate clinical improvement	
2	Seronegative juvenile polyarthritis	6	Young adulthood	NSAIDs, i/a and oral steroids, MTX, HCQ, leflunomide	Etanercept is under consideration for relapsing arthritis	None
3	AIHA	10	Young adulthood	IVIG	Remission after 9 yrs of treatment	Arthralgia, Hirschsprung disease, lymphoma, pneumonia, sepsis
4	Narcolepsy	11	Young adulthood	None specific, narcolepsy was not related to influenza vaccine	No remission	Allergy, anemia
5	AIHA	11	Died in adolescence	Prednisolone	Remission after short course of therapy	Anemia, pneumonia, sepsis
	Psoriasis	12		Local therapy	Disease under control until death	
	Enteropathy	14		None specific	Died from pneumonia following profound diarrhea	
6	Graves' disease	15	Young adulthood	Carbimazole, thyroidectomy	Cured after thyroidectomy	Allergy, arthralgia
7	ITP	29	Died in adulthood	Prednisolone	Remission after 1.5 yrs of therapy. Died from lymphoma	Allergy, arthralgia, diarrhea, lymphoma, pneumonia, sepsis
8	Celiac disease^*^	30	Died in adulthood	Unknown	Unknown. Died from pneumonia	Arthralgia, pneumonia
9	Enteropathy	35	Young adulthood	None specific	Investigations ongoing	Anemia, arthralgia, pneumonia
10	Psoriasis	41	Died in adulthood	Local therapy	Disease under control until death from lymphoma	Allergy, arthralgia, Hirschsprung disease, lymphoma, pneumonia
11	Ulcerative colitis	68	Died in mature adulthood	Mesalamine	Disease under control until death from end-stage lung disease	Allergy, asthma, bronchiectasis, meylodysplasia, pneumonia

*AI, autoimmune; AIHA, autoimmune hemolytic anemia; HCQ, hydroxychloroquine; i/a, intra-articular; ITP, idiopathic thrombocytopenic purpura; IVIG, intravenous immunoglobulin; MMAN, multifocal motor axonal neuropathy; MTX, methotrexate; No, patient number; NSAID, nonsteroidal anti-inflammatory drug; yrs, years*.

* *data on histopathology is not available*.

**Table 3:** Clinical and laboratory characteristics of 104 patients with cartilage-hair hypoplasia.

**Manifestations**	**No. of patients (%)**	**Comments**
Recurrent sinopulmonary and/or ear infections Hospitalization and/or surgery required	84/104 (81) 50/80 (63)	Acute otitis media, rhinosinusitis and/or pneumonia Tympanostomy and/or sinus surgery
Physician-diagnosed asthma	24/104 (23)	
Allergy to pollen and/or animal Positive allergy testing	40/104 (39) 5/9 (56)	Skin testing and/or allergen-specific serum IgE
Joint symptoms	56/104 (54)	Arthralgia or morning joint stiffness
Arthrosis Juvenile rheumatoid arthritis	20/56 (36) 1/56 (2)	Confirmed radiologically Seronegative polyarthritis
Gastrointestinal symptoms Prolonged and/or recurrent diarrhea Dyspepsia	32/104 (31) 23/32(72) 18/32 (56)	
Gastroscopy performed Colonoscopy performed Chronic gastritis Duodenal villous atrophy	18/32 (56) 8/32 (25) 5/18 (28) 3/18 (17)	Normal results in eight (8/18, 44%) patients Normal in all except one case of ulcerative colitis Diagnosed on gastroscopy Diagnosed on gastroscopy
Anemia Red blood cell transfusion required AIHA	32/104 (31) 13/32 (41) 2/32 (6)	
Elevated IgE^*^	5/69 (7)	
Elevated deamidated gliadin peptide IgA	2/33 (6)	Values of 9 and 20 (normal < 7 U/ml)
Elevated deamidated gliadin peptide IgG	2/33 (6)	Values of 10 and 14 (normal < 7 U/ml)
Elevated interferon ω IgG	1/33 (3)	Value of 206 (normal < 200 Index)
Elevated 21-Hydroxylase IgG	2/33 (6)	Values of 67 and 77 (normal < 57 Index)

*AIHA, autoimmune hemolytic anemia; Ig, immunoglobulin; No, number*.

* *According to local laboratory age-adjusted reference values*.

Among those with autoimmunity, recurrent pneumonia, and sepsis were more frequently documented than in those without autoimmunity (6/11, 55% vs. 13/93, 14%, *p* = 0.001, B coefficient 2.65 and 4/11, 36% vs. 6/93, 6%, *p* = 0.019, B coefficient 2.14, respectively). Rates of autoimmunity were higher in patients with elevated IgE levels (2/5, 40%; *p* = 0.011, B coefficient 2.96) and in patients with undetectable IgA levels (3/5, 60%; *p* = 0.036, B coefficient 3.33), as compared with patients with normal IgE and IgA levels.

**Figure 1:**
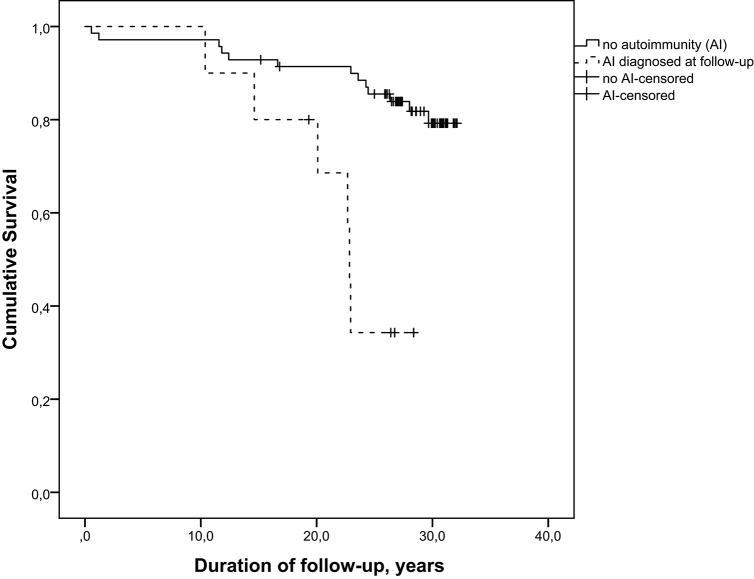
Survival of patients with cartilage-hair hypoplasia differs significantly in subjects with and without autoimmune conditions, log rank $\chi_{\left(2\right)}^{2}$ = 14.056, *p* = 0.0002.

Among patients from Cohort 1, 10/80 individuals have developed AI diseases during follow-up. The Kaplan-Meier analysis (log rank test) of these 80 patients demonstrated that survival distributions for patients with and without AI conditions were significantly different, $\chi_{\left(2\right)}^{2}$ = 14.056, *p* = 0.0002 (Figure 1).

**Figure 2:**
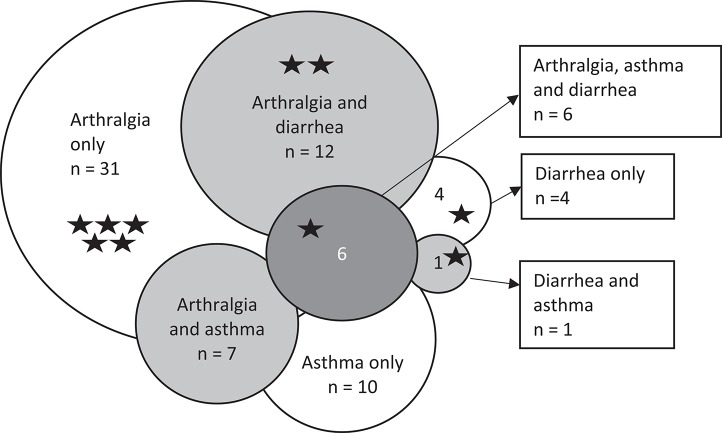
Modified Venn diagram of arthralgia, asthma, persistent diarrhea and their combinations in 71 patients with cartilage-hair hypoplasia. *n*, number of patients. Stars indicate patients with autoimmune diseases.

More than half of our patients (56/104, 54%) suffered from arthralgia or morning joint stiffness, but only one was diagnosed with genuine seronegative juvenile polyarthritis. Osteoarthritis was common among patients with arthralgia (20/56, 36%) and was increasingly prevalent with increasing age. Mean age at last follow-up in those with osteoarthritis was 51.7 years, while it was 35.2 years in those without osteoarthritis. Patients with a history of arthralgia reported more often sinusitis (38/56, 68% vs. 18/48, 38%, *p* = 0.002, B coefficient 1.3) and pollen allergy (27/56, 48% vs. 13/48, 27%, *p* = 0.035, B coefficient 0.95). When arthralgia, asthma and diarrhea were compiled in a Venn diagram (*n* = 71), asthma and diarrhea overlapped with arthralgia in 7 and 12 patients, respectively (Figure 2). The overlap, however, did not reach statistical significance. This group of 71 patients contained most of the patients with AI conditions (10/11).

Gastrointestinal symptoms were common in our cohort (32/104, 31%) and the most frequent complaint was prolonged/recurrent diarrhea. In at least three patients with diarrhea, broad screening for bacterial, viral and parasitic infections was negative. Twelve patients followed lactose-free diet, but this did not alleviate diarrhea. Eight patients had undergone colonoscopy, with normal results in all except one subject with ulcerative colitis. Gastroscopy findings were abnormal in 10/18 patients and included gastroesophageal reflux (*n* = 5), chronic gastritis (*n* = 5), peptic ulcer (*n* = 2), and/or duodenal villous atrophy (DVA, *n* = 3). Another patient had received the diagnosis of celiac disease, but data on histology were unavailable. Significantly more patients with diarrhea reported arthralgia (18/23, 78% vs. 38/81, 47%, *p* = 0.011, B coefficient 1.41), as compared with those without diarrhea. In the subgroup of patients with osteoarthritis (*n* = 20), diarrhea was not more common than in those without osteoarthritis.

Anemia was diagnosed in 32 patients (31%), mostly in childhood. Thirteen of these patients (41%) required red blood cell transfusions. Two subjects had AIHA, in others anemia was hypoplastic and attributed to CHH itself and mostly resolved by adulthood. Four of the patients required blood transfusions as adults. There were no cases of AI neutropenia, but one patient had idiopathic thrombocytopenic purpura.

Physician-diagnosed asthma (23%) and allergy (39%) were both highly prevalent. Asthma was diagnosed in 29% (5/17) of children and in 22% (19/87) of adults. In some patients with clinical symptoms of pollen allergy, skin testing and/or serum allergen-specific IgE testing had been negative. Elevated IgE levels were infrequently documented (5/69 patients), also in subjects without allergic symptoms. Eosinophilia was more common in subjects with pollen allergy (5/34, 15% vs. 1/50, 2%, *p* = 0.038). Hospitalization for pneumonia and/or surgery for sinopulmonary and/or ear infections were more common in patients with asthma [19/24 (79%) vs. 31/80 (39%), *p* = 0.019, B coefficient 1.13].

### Subgroup of patients tested for autoantibodies

Serum samples were available from 33 adult patients (21 women, 12 men) at median age of 45.4 years (range 19.9–69.1 years). One patient was receiving immunoglobulin substitution at the time of blood sampling, he tested negative for all autoantibodies. One had unmeasurable IgA, therefore EMA-IgG and tTG-IgG were measured in serum with normal results. In a few patients, we detected slightly elevated autoantibodies including anti-interferon-ω IgG (IFN-ω, *n* = 1), anti-21-hydroxylase IgG (21-OH, *n* = 2), anti-gliadin-peptide IgA (*n* = 2) and anti-gliadin-peptide IgG (*n* = 2; Tables 3, 4). These patients had no celiac disease and none of the typical manifestations of APECED, including adrenal insufficiency, hypoparathyroidism and mucocutaneous candidiasis. All other autoantibodies tested negative in all patients.

**Table 4:** Results of autoantibody measurements in 33 patients with cartilage-hair hypoplasia.

**Pt No**.	**AI**	**Arthralgia**	**G/i**	**Pollen allergy**	**BA**	**DGP-IgA, U/ml**	**DGP-IgG, U/ml**	**tTG-IgA, U/ml**	**EMA-IgA, titer**	**IL-17, index**	**IL-22, index**	**IFN-ω, index**	**21-OH, index**
1	–	+	–	+	+	<7	<7	<7	<5	211	−52	34	23
2	–	–	–	–	–	<7	**14**	<7	<5	−5	81	64	55
3	–	+	–	+	–	**20**	**10**	7	<5	−2	−9	62	−27
4	–	+	–	+	–	<7	<7	<7	<5	64	2	−50	−34
5	–	–	–	+	–	<7	<7	<7	<5	−49	−25	0	6
6	–	+	+	–	–	7	7	<7	<5	−37	30	13	40
7	–	+	+	+	–	<7	<7	<7	<5	−30	−70	111	11
8	–	+	+	+	–	<7	<7	<7	<5	31	101	155	35
9	–	+	+	+	–	<7	<7	<7	<5	−28	−7	49	−33
10	–	+	+	+	+	<7	<7	<7	<5	−53	84	49	47
11	–	+	+	–	–	<7	<7	<7	<5	−62	23	−39	−21
12	–	–	+	+	+	<7	<7	<7	<5	−40	90	136	**67**
13	–	+	–	+	+	<7	<7	<7	<5	−84	−95	−48	−30
14	–	+	–	+	–	<7	<7	<7	<5	−27	99	22	15
15	–	–	–	–	–	<7	<7	<7	<5	−10	−60	171	46
16	–	–	–	–	–	<7	<7	<7	<5	−32	39	29	11
17	–	+	–	+	–	<7	<7	<7	<5	−32	73	48	77
18	–	–	+	+	–	<7	<7	<7	<5	−62	−11	−46	7
19	–	+	–	+	+	<7	<7	<7	<5	−52	−110	−110	−4
20	–	+	+	–	–	**9**	<7	<7	<5	−42	−27	−86	−21
21	–	–	–	–	–	<7	<7	<7	<5	−26	15	−36	46
22	–	+	–	–	–	<7	<7	<7	<5	16	105	130	**77**
23	–	–	+	–	–	<7	<7	<7	<5	−59	50	79	54
24	–	–	–	+	–	<7	<7	<7	<5	−66	3	54	16
25	–	–	–	–	–	<7	<7	<7^*^	<5^*^	51	37	68	18
26	–	+	–	–	–	<7	<7	<7	<5	−38	−10	42	21
27	–	–	–	–	–	<7	<7	<7	<5	48	183	−194	−4
28	–	–	–	–	–	<7	<7	<7	<5	−98	−71	−71	−1
29	–	+	+	–	–	<7	<7	<7	<5	−69	−18	−45	4
30	–	–	+	+	+	<7	<7	<7	<5	−69	19	−202	−30
31	–	–	–	–	–	<7	<7	<7	<5	−8	66	**206**	38
32	–	–	+	+	–	<7	<7	<7	<5	−32	90	69	30
33	–	+	+	+	–	<7	<7	<7	<5	−51	−17	40	40

*Numbers in bold represent elevated values. For reference values please see Table 1*.

* *Because of immunoglobulin A deficiency diagnosed in this patient, tTG-IgG and EMA-IgG were measured, both were negative. 21-OH, 21-hydroxylase; AI, autoimmune disease; BA, bronchial asthma; DGP, deamidated gliadin peptide; EMA, endomysium; G/i, gastrointestinal symptoms; IFN, interferon; Ig, immunoglobulin; IL, interleukin; No, number; Pt, patient; tTG, tissue transglutaminase*.

### Subgroup of patients tested for nasal cytology

Nasal samples were available from five patients. Although they all reported physician-diagnosed allergic rhinitis, eosinophils were almost absent in all subjects, only two out of five subjects had single cells visible unilaterally (Table 5). Further, neutrophils were detected in moderate amounts in only one patient, and goblet cells in another. Lymphocytes were not found in any samples.

**Table 5:** Cytology of nasal cells from five subjects with cartilage-hair hypoplasia.

	**Subject 1**		**Subject 2**		**Subject 3**		**Subject 4**		**Subject 5**	
**Symptoms**	**Pollen allergy**, **rhinoconjuctivitis**		**Pollen allergy**		**Pollen allergy, nasal congestion**, **shortness of breath and wheezing**		**Chronic nasal** **congestion**, **runny nose**		**Periodic nasal** **congestion**	
Nasal side	Right	Left	Right	Left	Right	Left	Right	Left	Right	Left
Lymphocytes	–	–	–	–	–	–	–	–	–	–
Eosinophils	–	(+)	–	–	–	–	(+)	–	–	–
Neutrophils	–	+	–	+	–	–	(+)	(+)	++	++
Goblet cells	++	++	(+)	(+)	–	–	–	–	(+)	(+)

*Grading of eosinophilic cells (per microscopic view): –, 0 cells; +/-, 1-3 cells; +, 3–5 cells; ++, 20–30 cells; +++, cells predominate. Grading of neutrophilic cells (per microscopic view): –, no cells; +, cells clearly visible; ++, moderate number of cells visible*.

## Discussion

We demonstrate high prevalence (11/104, 10.6%) of AI conditions in a large cohort of Finnish patients with CHH compared with the prevalence of 5.4% in general Finnish population (23). Autoimmunity, allergy, diarrhea, arthralgia, and asthma in our patients may reflect common pathways of immune dysregulation behind these symptoms.

With microarray technique, multiple autoantibodies have been demonstrated in all (*n* = 16) tested serum samples from patients with CHH (15). The absence of compatible symptoms may reflect the benign nature of these antibodies, consistent with reported presence of autoantibodies in healthy individuals (24). *RMRP* mutations may contribute to AI phenomena by various mechanisms, such as impaired T_h_17 cell functions or altered expression of *AIRE*. Autoantibodies typically observed in APECED patients (IFN-ω, IL-17, IL-22, 21-OH) have not been included in our previous study. Here, we describe positivity for these antibodies in three of our patients, but the indices were close to the limit of positivity and the patients had no compatible clinical manifestations. Thus, the significance of these findings is uncertain.

The data on the CD4+CD25^high^CD127^low^ regulatory T cells measured in 11 patients from our cohort has previously been published (6). The numbers of regulatory T cells were normal in 10/11 patients, including two patients with autoimmunity (measured after the development of AIHA and juvenile idiopathic arthritis) and low in a single 67-year-old patient without AI manifestations. However, functional abnormalities in regulatory T cells cannot be excluded and the role of CTLA-4 deficiency behind the immune dysregulation should be further explored.

Many of our study patients described gastrointestinal problems, most commonly recurrent and/or prolonged diarrhea. Eleven of our patients avoided lactose, which did not improve diarrhea. Endoscopy was not systematically performed in patients with CHH and thus AI enteropathy cannot be excluded. The presence of DVA in three subjects with negative celiac screen underscores the necessity for systematic endoscopic evaluation of CHH individuals with gastrointestinal complaints. Apart from celiac disease, DVA is frequent in patients with common variable immunodeficiency and numerous other disorders, and DVA etiology should always be identified and treated to prevent malnutrition and malignant transformation (25, 26).

Serum tissue transglutaminase (tTG) IgA was negative in all tested patients and tTG IgG was negative in a single patient with IgA deficiency. However, in our previous microarray study (15), five out of 16 patients tested positive for tTG IgG and two of them reported diarrhea, therefore the possible link between tTG antibodies and gastrointestinal symptoms in patients with CHH deserves further studies. Isolated positive tTG IgG is uncommon in celiac disease, but can be present in other AI conditions, including inflammatory bowel disease (27). The discordance of positivity for tTG IgG in our current and previous study may be explained by higher sensitivity of microarray technique.

Asthma was more common in individuals with CHH who required hospitalization and/or surgery for sinopulmonary and/or ear infections. This may suggest asthma as a marker for a more severe immunodeficiency in CHH but may also reflect the retrospective nature of our study and the influence of age, with accumulation of infectious episodes and asthma diagnoses in older patients. Asthma was not associated with pollen allergy in this cohort, raising the question of possible misdiagnosis of one or both of these disorders in some patients. Asthma diagnosis is challenging in CHH due to the absence of height-age-adjusted reference values for spirometry.

Autoimmunity has been implied as a mechanism for lung disease in some patients with APECED and asthma-like symptoms, airway hyperresponsiveness and bronchiectasis (28, 29). We have previously demonstrated high prevalence of bronchiectasis in individuals with CHH, also in those without clinical signs of immunodeficiency (5). Thus, some patients with CHH designated to have asthma, may instead suffer from an underlying autoimmune lung disease and pulmonary problems in subjects with CHH require further investigations.

Four of the eleven patients with AI diseases died of pneumonia or end-stage lung disease. Although in three of them lung manifestations preceded the onset of autoimmunity, we suggest that patients with CHH and AI conditions should be carefully evaluated for pulmonary symptoms and consideration given for prophylactic antibiotics and/or immunoglobulin substitutions to prevent lung damage from recurrent infections.

In allergic rhinitis and asthma, eosinophils are recruited by pro-inflammatory cytokines and mediators and are the predominant cells in later phases of an allergic response (30, 31). The role of neutrophils in allergic inflammation is debated, but some studies report increasing numbers in nasal cytology in patients with allergic rhinitis (32–34). While all five tested subjects reported recurrent or chronic symptoms of allergic rhinitis and nasal samples were collected during the peak pollen season, none had increased numbers of eosinophilic, neutrophilic or lymphocytic nasal cells. This points to the presence of a chronic mucous inflammation, congruent with the systemic immune dysregulation, also supported by the fact that some patients with symptoms of pollen allergy tested negative for allergens.

In conclusion, we report here a high prevalence and a wide spectrum of AI diseases in a large cohort of patients with CHH. We report mild positivity for some autoantibodies, not associated with clinical autoimmunity. The high prevalence of arthralgia, persistent diarrhea, asthma and symptoms of inflammation of nasal mucosa may indicate common pathways of immune dysregulation. Gastrointestinal, pulmonary and joint symptoms in subjects with CHH should be thoroughly evaluated to exclude underlying autoimmunity.

## Ethics statement

This study was carried out in accordance with the recommendations of Institutional Research Ethics Committee at Helsinki University Hospital, Finland, with written informed consent from all subjects. All subjects gave written informed consent in accordance with the Declaration of Helsinki. The protocol was approved by the Institutional Research Ethics Committee at Helsinki University Hospital, Finland.

## Author contributions

OM, SV, and EH designed the study; SV analyzed the data and drafted the manuscript; RM performed nasal sampling; EH performed autoantibody analysis. All authors contributed to the manuscript writing and approved the final version.

### Conflict of interest statement

The authors declare that the research was conducted in the absence of any commercial or financial relationships that could be construed as a potential conflict of interest.

## Abbreviations

AI: autoimmune
AIHA: autoimmune hemolytic anemia
APECED: Autoimmune Polyendocrinopathy-Candidiasis-Ectodermal Dystrophy
CHH: cartilage-hair hypoplasia
DVA: duodenal villous atrophy
Ig: immunoglobulin
IL: interleukin
RMRP: RNA component of mitochondrial RNA-processing endoribonuclease.
